# What Is the Evidence for Paediatric/Adolescent Bariatric Surgery?

**DOI:** 10.1007/s13679-017-0277-4

**Published:** 2017-08-16

**Authors:** Natalie Durkin, Ashish P. Desai

**Affiliations:** Department of Paediatric Surgery, King’s College Hospital, Denmark Hill, London, SE9 5RS UK

**Keywords:** Paediatric, Bariatric surgery, Adolescent, Benefits, Complications, Sleeve gastrectomy

## Abstract

**Purpose of Review:**

In spite of the increasing prevalence of severe and complex obesity in children, surgery as a potential management option is still not widely accepted. The purpose of this review is to examine the evidence for surgical options in the severely obese paediatric population. Increasing evidence supports early rather than later use of bariatric surgery in the treatment of extreme obesity.

**Recent Findings:**

Prior to 2007, the feasibility and safety of surgery have been reported by predominantly small, sporadic single-centre retrospective case series. Increasing long-term data is now emerging due to the formation of multi-centre prospective national consortiums with two large, prospective long-term outcome studies published within the last year aiding our understanding of the efficacy and safety of bariatric surgery within the adolescent population.

**Summary:**

It is increasingly clear that adolescent bariatric surgery outcomes are comparable to adults, with similar sustainable weight loss, resolution of co-morbidities and complication rates. However, these studies are solely from dedicated specialist adolescent centres and results may not be reproducible if not performed in regulated environments with specialist multi-disciplinary teams.

## Introduction

Obesity is a global epidemic. Childhood obesity in particular has become an increasing concern due to the rising prevalence of associated co-morbidities including metabolic syndrome and diabetes, obstructive sleep apnoea syndrome (OSAS) and psychosocial impairments, at ever-younger ages. These co-morbidities have a cumulative health impact, making the duration of obesity increasingly important and predisposing patients to a significant risk of premature morbidity and mortality [1]. As such, childhood obesity is fast becoming the most significant threat to the health of our younger generations.

Lifestyle interventions have been shown to have limited success in treatment of severe obesity in children. In adults, sustained BMI reduction and significant and definitive risk reductions for developing cardiovascular, cancer, endocrine, infectious and psychiatric disorders have been found in surgical cohorts as compared to controls [2]. With such life-changing results in the adult population, why not, then, apply the same intervention prior to the development of complications of co-morbidities in the paediatric population? This article aims to explain the rationale for paediatric bariatric surgery, review the growing evidence base of outcomes and explore the controversies that still exist in order to delineate the future of this growing problem.

## Prevalence of Childhood Obesity

Defining obesity in children presents slightly different challenges to that in adults. The body mass index (BMI) provides a relatively good gauge of body fat, whilst being easy to measure and subsequently calculate. As such, standard levels of BMI classify obesity in adults; if the BMI > 30 kg/m^2^, this is considered obese. Children have varying proportions of body fat at varying ages which also differs depending on gender. As such, fixed levels of BMI provide inaccurate comparisons.

In children, gender and age-specific BMI growth charts of large reference populations consider the pattern of growth over time. BMI Z-scores/standard deviation score (BMI-SDS), a measure of how many standard deviations a child’s BMI is above the mean, are used to provide set definitions of obesity. The most widely used definition of ‘severe’ obesity is a BMI > 99th centile, broadly equivalent to a BMI Z-score of +2.5, an adult BMI equivalent 30 kg/m^2^ [3]. Adolescent BMI > 99th centile has specifically been shown to have a strong positive correlation with adverse cardiovascular risk profile [1].

Prevalence of childhood obesity has risen by 47.1% between 1980 and 2013. Although the overall proportion of childhood obesity appears to have plateaued out over the last decade, there is no convincing evidence of a sustainable decline. The National Child Measurement Programme (NCMP) in the UK identified 21.9% children aged 4–5 were classified as overweight or obese, a figure, which rose to one third of 10–11-year-olds [4]. Based on a definition of a BMI > 99th centile, 2.9% of girls and 3.9% of boys in the UK have severe obesity [5]. This finding is paralleled in other developed countries with the prevalence of severe obesity affecting approximately 4% of US adolescents [6]. This is an effect now seen in developing countries with a 60% increase in prevalence in recent years [7]. Childhood obesity is, therefore, a truly global phenomenon of growing concern.

## Effect of Lifestyle Intervention as Treatment Modality

Community-based interventions have yet to demonstrate significant and sustainable results. A 2009 Cochrane review of combined behavioural and lifestyle management of paediatric obesity in 5230 patients as compared to controls demonstrated a − 0.06 overall reduction of BMI-SDS in the under 12-year-olds, with only a slight improvement of −0.47 BMI-SDS in a more recent systematic review in 2014 [8, 9]. A decrease in BMI-SDS of <−0.25 was found in <10% of the group. In addition, children with a lower BMI (30–35) achieved better results than those deemed to more extreme obesity (BMI 35+), indicating lifestyle intervention is more effective in those with lower BMI scores.

## Rationale for Surgery

As discussed, lifestyle modification alone does not appear to achieve substantial and sustainable weight loss in obese adolescents. As far back as 1993, Must et al. studied the long term relationship between overweight and obese adolescents and cardiovascular morbidity and found increased mortality with coronary heart disease in men [10]. Similar increases in all-cause mortality have since been demonstrated in another study of 1.46 million white adults [11]. Failure of lifestyle management, increased mortality for untreated patients and early results of adolescent surgery have all indicated that adolescent bariatric surgery is a successful therapeutic option if lifestyle modifications fail.

## Eligibility Criteria

There are multiple guidelines regarding criteria for surgery. Aikenhead et al. reviewed current guidelines worldwide and suggested that although they were all similar, they lacked uniformity regarding the age and severity of obesity at which intervention should be offered [12]. NICE (National Institute for Health and Clinical Excellence, UK) suggests that the surgery should only be carried out in this age group under exceptional circumstances, however, recommends following the same BMI criteria as adults [13]. The international paediatric endosurgery group (IPEG) recommends surgery in adolescents who have attained or almost attained adult stature with specific guidance (see Table 1) [14]. The unifying factor in all guidelines appears to be recommending the use of multi-disciplinary team (MDT) and the need for long-term follow-up.

**Table 1:** Adolescents being considered for bariatric surgery should fulfil all of the below criteria

✓ Be very severely obese (BMI ≥ 40) with serious obesity related co-morbidities ✓ Have attained or depending on the severity of co-morbidity, nearly attained adult stature ✓ Have failed at least 6 months of organised conventional attempts at weight management ✓ Demonstrated commitment to comprehensive paediatric psychological evaluation both before and after surgery and agree to avoid pregnancy for at least 1 year post-operatively ✓ Be capable of and willing to adhere to nutritional guidelines post-operatively ✓ Have decisional capacity and provide informed assent for surgical management.
Comorbid conditions • Serious comorbidities • Type 2 diabetes mellitus • Obstructive sleep apnoea • Pseudotumor cerebri
Less serious comorbidities • Weight related arthropathy • Hypertension • Dyslipidaemia • Venous stasis disease • Panniculitis • Urinary incontinence • Significant impairment in activity of daily living • Non-alcoholic fatty liver disease (includes steatohepatitis) • Gastroesophageal reflux • Severe psychosocial distress

Available at https://www.ipeg.org/morbidobesity/. Copyright ©International Paediatric Endosurgery Group (IPEG), used with permission

There is no specific evidence for the use of a multidisciplinary team either for adults or adolescents undergoing bariatric surgery. Considering the complex nature of intervention, however, particularly at the vulnerable age of these patients, the MDT has become well established as the gold standard of care necessary to provide a safe and efficient service.

The team members recommended by the American Society of Metabolic and Obesity Surgeons (ASMBS) in 2012 [15] includes the following:

1. An experienced bariatric surgeon
2. A paediatric specialist; either a paediatrician with a specialty in endocrinology, gastroenterology, nutrition, and/or adolescence or an internist or family practitioner with training in adolescent medicine.
3. A registered dietician with experience in treating obesity and working with children and families.
4. A mental health specialist; a psychiatrist or psychologist with specialty training in paediatrics +/− adolescents and particular experience in treating eating disorders and obesity. In addition, the practitioner should have experience evaluating patients and families for bariatric surgery.

Further recommendations, although not essential, include a coordinator and an exercise physiologist or physical therapist to provide safe physical activity prescriptions to morbidly obese adolescents. Teams should follow up patients for at least for 2 years after surgery. Transition arrangements should be finalised with adult teams however as far as the authors are aware, there are no published guidelines about this.

## Outcomes

### Weight Loss

Evidence within the adult population demonstrates clear justification for bariatric surgery with regard to sustainable weight loss and improvement of co-morbidities. Chang et al. published a systematic review and meta-analysis of outcomes in 161,756 adult patients undergoing bariatric surgical procedures in 2014. Both Roux-en-Y gastric bypass (RYGB) and laparoscopic sleeve gastrectomy (LSG) were shown to be effective in achieving weight loss with BMI loss of −15.9 and −16.1 kg/m^2^, respectively, at 5 years. The complication rate was 17% with a 7% reoperation rate and a 0.08% <30-day mortality. Obesity-related co-morbidities were found to resolve almost universally with remission of diabetes (92%), hypertension (75%), dyslipidaemia (76%) and OSA (96%) [16].

Despite such a wealth of adult evidence, there has been very little in the way of comparable studies from the adolescent population. Black et al. published a meta-analysis of adolescent bariatric surgical articles including 637 patients from 23 studies. The mean BMI change was −13.5 kg/m^2^ for combined procedures (RYGB, LSG, LAGB) with the largest drop in the RYGB group at 1 year (−17.2 kg/m^2^) [17•]. The studies included had significant heterogeneity and as such outcomes with regard to resolution of co-morbidities and complications were inconsistently reported.

This heterogeneity is partly because prior to 2007 paediatric outcome data was sparse with predominantly small, retrospective, single-centre case series with different definitions and end points. There has been a real drive from the international community to quantify outcomes within this population. Three main centres have published extensively with a change in approach from retrospective to prospective collection of outcomes with results detailed below. Additionally, within the last year, the first set of prospective long-term (>5 years) outcomes have been published by both the adolescent morbid obesity surgery (AMOS) and Teen-Longitudinal Assessment of Bariatric Surgery (Teen-LABS) consortiums providing an insight into the sustainability of weight loss, long-term effects on co-morbidities and the safety of bariatric surgery in childhood.

In 2012, Alqahtani et al. published the first large 3-year single-centre retrospective review of 108 paediatric patients (mean age 13.9+/−4.3 years) compared to a non-matched cohort of 114 adult patients. This demonstrated comparable percentage excess weight loss (EWL) at 2 years between the two groups (64.9 vs 69.7%, respectively), despite a much higher mean BMI in the paediatric group (49.6 vs 32.2 kg/m^2^) [18••]. The post-operative complication rate was 5.6%. Follow-up work of an increased cohort of 226 adolescent patients (mean 14.4 +/−4) undergoing LSG in 2014 demonstrated remission of 90.3% of co-morbidities including obstructive sleep apnoea, hypertension, diabetes and dyslipidaemia at 2 years. Importantly, although one third of all patients within this cohort were under 12 years old, all patients experienced normal growth velocity [19•].

The Teen-LABS consortium was established in 2007 as a prospective, multi-centre observational study to measure efficacy and safety of weight loss interventions. The key to analysis was standardising management protocols, definitions and methodology. In 2014, this group published the largest series of prospectively collected data on 242 adolescent patients with a mean age of 17.1 (+/−1.6) and BMI of 53 [20•]. Mean weight of patients decreased by 28% with gastric bypass and 26% with sleeve gastrectomy, which was sustained at 3 years. Convincing improvements were made in obesity-related co-morbidities; remission of diabetes was seen in 95% of patients, with similar improvements in those with abnormal kidney function (86%), dyslipidemia (66%) and hypertension (74%). During the 30-day post-operative period, 8% of patients suffered a major complication and 15% defined as minor. There were no mortalities. Over 3 years follow-up, 13% underwent one or more additional intra-abdominal procedures [21]. Since 2014, the Teen-LABS consortium has consistently published prospective results of subgroups of their cohort with regard to prevalence and resolution of obesity-associated co-morbidities that will be later discussed.

The AMOS study is a Swedish multi-centre consortium producing prospective, non-randomised controlled studies, the first of which was initially published in 2012 [22•]. Outcomes of RYGB in 81 adolescents (mean age 16.5 (+/−1.2)) were compared with gender, age and BMI-matched controls. At 2 years follow-up, mean weight loss post-operatively was −32% in the surgically treated adolescent group vs +3% weight gain in the conventionally treated controls. The surgically treated adolescents were also compared to an adult cohort of gender and BMI-matched patients who achieved comparable weight loss (−31%). A follow-up paper was published in March 2017 representing the largest 5-year follow-up study of adolescents undergoing RYGB to date [23••]. Of the adolescent surgical cohort, they had an impressive 100% follow-up rate at 5 years. Despite some limitations to the study, namely a non-randomised control arm with non-standardised conservative treatment and a 25% cross-over to the operative arm, the authors were still able to demonstrate comparative 5-year weight loss to the 2-year results, compared with ongoing weight gain in the adolescent control group. Significant long-term improvements in co-morbidities were also seen in the surgical group with 100% resolution of type 2 diabetes and hypertension, 82% resolution of dyslipidaemia and normalisation of liver function tests in 92%.

In March 2017, Inge et al. published the results of the US prospective cohort study, follow-up after bariatric surgery (FABS-5). Fifty-eight patients undergoing RYGB between 2001 and 2007 were followed up to analyse long-term outcomes with a retention rate of 81% [24••]. The mean baseline age was 17.1 (+/−1.7) years with a mean BMI of 58.5 kg/m^2^. Published 1-year BMI reduction was −22.8 kg/m^2^ and at a mean follow-up of 8 years, this weight loss was almost entirely maintained at a mean of −16.9 kg/m^2^ from baseline BMI at time of operation. In addition, a statistically significant ongoing decline in the prevalence of metabolic syndrome was noted with hypertension found in 19 vs 47% at operation, dyslipidaemia in 38 vs 86% and type 2 diabetes in 2 vs 16%. Overall in this cohort, 63% of participants remained obese (BMI >35 kg/m^2^) at follow-up. In this study, Inge et al. further reinforced previous conclusions that there is a strong positive correlation between BMI at baseline and BMI at long-term follow-up. This continues to indicate that patients operated on earlier, at relatively lower BMIs, may achieve more successful weight loss post-surgery to normalise BMIs to non-obese levels than that seen in those with higher initial BMI.

## Resolution of Co-morbidities

Increased paediatric obesity has also seen rise in the prevalence of obesity-related co-morbidities at ever younger ages, affecting almost all organ systems. It has long been recognised that obese children are at a high risk of metabolic syndrome, a constellation of glucose resistance, hypertension and hypercholesterolemia. Regression modelling from the FABS-5+ study demonstrated that there is also a significant proportional relationship between BMI and cardiometabolic risk. For every 10 kg/m^2^ increase in BMI, there was a 34% increase in dyslipidaemia risk, 46% higher risk of hypertension and 25% rise in insulin concentration. Because the cardiovascular complications of metabolic syndrome result from cumulative years of exposure and subsequent atherosclerosis, earlier onset of obesity equates to an increased risk of premature death from cardiovascular disease. A retrospective review of 2.3 million Israeli adolescents (mean age 17.3+/−0.4) by Twig et al. aimed to quantify the risk of cardiovascular death in adulthood over 43 years [25]. Hazard ratios for the obese adolescent group (BMI >95th centile) as compared to the 24th centile were 4.9 for death from coronary heart disease, 2.6 for stroke and 3.5 for death from total cardiovascular causes. Adult literature has clearly demonstrated improvement in individual cardiovascular risk factors after bariatric surgery, but also a clear overall reduction of the risk of stroke, myocardial infarction and death by approximately 50% [26]. Although there are no similar long-term studies in the adolescent population as yet, both the FABS-5+ and AMOS cohorts have demonstrated ongoing long-term regression of hypertension, dyslipidaemia and diabetes in the majority of patients. Ippisch et al. also quantified the reversibility of cardiac abnormalities in adolescents (mean age 16+/−1 years) pre and post-bariatric surgery. Echocardiography found that predictors of future cardiovascular morbidity including the left ventricular mass, hypertrophy, diastolic function and cardiac workload all significantly improved following surgically induced weight loss in 38 adolescents over 10 months [27]. It would therefore be logical to suggest that cardiovascular morbidity and mortality should decrease in severely obese adolescents undergoing bariatric surgery.

The prevalence of type 2 diabetes mellitus (T2DM) in young people has risen significantly over the last 25 years following the rise in childhood obesity. Whilst in 1992, it accounted for 3% of all new cases of diabetes in children [28]; it currently accounts for almost 50% [29]. The associated complications affect several organ systems with severity directly related to length of disease due to a cumulative effect. Other than a significantly increased cardiovascular risk, T2DM in adults is responsible for more cases of renal failure and peripheral vascular disease leading to amputation than any other disease [30]. Adolescent type 2 diabetes also appears to be a more aggressive form with renal complications developing much earlier compared with type 1 diabetes; 6% of adolescents develop renal failure by age 20 years, and by 29 years of age, 2.3% have developed end-stage renal disease [31]. There also appears to be a more rapid progression to insulin requirements in adolescent T2DM when compared to type 1 [32•].

RYGB has been shown to increase insulin sensitivity by four times in adolescents both with and without diabetes after surgery [33]. Five-year remission rates of diabetes post adolescent surgery in the AMOS and FABS-5+ cohorts are at 100 and 88%, respectively, although numbers were small (*n* = 9, *n* = 3). There appears to be a greater efficacy in remission of diabetes after bariatric surgery in adolescents compared to adults, possibly due to a shorter duration of obesity and reduced severity of disease at presentation. Panunzi et al. combined data from the Swedish Obesity Subjects (SOS) trial and two randomised controlled studies in adults and found shorter diabetes duration and lower fasting glucose prior to surgery independently predicted significantly higher rates of remission [34]. This may be a further contributory factor suggesting we should be operating on adolescents earlier.

Non-alcoholic fatty liver disease (NAFLD) is strongly associated with obesity and is considered a spectrum of pathology ranging from NAFLD to non-alcoholic steatohepatitis (NASH), with a 20% 10-year progression to cirrhosis and fibrosis. With the increasing prevalence of obesity, NASH is currently the primary cause of liver function abnormalities and chronic liver disease in children [35] and is predicted to be the most common indication for liver transplantation in the next 10 years [36]. Of 148 adolescents undergoing bariatric surgery in the Teen-LABS cohort, 59% had biopsy evidence of NAFLD with mild fibrosis seen in 18% [37]. An adult meta-analysis by Muhammadi et al. showed improvement or resolution of steatosis in over 90% of patients after bariatric surgery [38]. No such outcome data exists in children; however, the AMOS study did demonstrate a 92 and 100% resolution of alanine aminotransferase and aspartate aminotransferase to normal levels at 5-year follow-up post-bariatric surgery. This does raise the question whether earlier intervention in adolescents not only prevents progression from NAFLD to NASH but also leads to normalisation of inflammation and fibrosis in patients who already have NASH at the time of surgery [39].

Obesity-associated respiratory pathologies including obstructive sleep apnoea have both immediate consequences of poor school performance and irritability alongside long-term consequences such as sustained nocturnal hypertension, left ventricular hypertrophy and increased cardiovascular and cerebrovascular morbidity and mortality [40, 41]. Adult studies have demonstrated the usefulness of bariatric surgery in improving OSA outcomes with a systematic review of 1350 patients demonstrating greater than twice the reduction in apnoea-hypopnoea index in surgical patients vs non-surgical controls [42]. Of 19 adolescents undergoing RYGB with pre-operative obstructive sleep apnoea, Kalra et al. saw a marked decrease in severity of OSAS in *all* patients undergoing post-operative polysomnograms, although they were only able to follow up 53% [43]. Further work by Amin et al. in a small, prospective study of seven adolescents undergoing surgery demonstrated a 40% reduction in apnoea score at 5 weeks compared to controls [44].

The consequences of obesity on psychosocial well-being are also not to be underestimated. Multiple studies have demonstrated significant reductions in global self-esteem and quality of life in obese youth and adolescents seeking weight loss surgery have been shown to have higher levels of depressive symptoms [45–47]. These children have also been found to have lower attainment in education and training and are more likely to not complete education, which may be partly attributable to the frequency of weight-based victimisation and stigma [48, 49]. The AMOS collaboration demonstrated a substantial improvement in psychosocial well-being in adolescents 2 years post-gastric bypass vs controls. Symptoms of anxiety, depression, anger and disruptive behaviour were significantly reduced (*p* = 0.001) and self-esteem, self-concept and overall mood significantly improved (*p* < 0.001) [50]. However, clinically depressive symptoms were found in 19% at 2 years, with two cases of attempted suicide. The Teen-LABS consortium has also demonstrated variable mental health outcomes with surgery. Of 11 patients presenting with ≥1 mental health symptom pre-surgery, remission was only found in 45% [51], although no new cases developed. These findings suggest that not all adolescents benefit psychologically from bariatric surgery, and the role of psychological screening prior to operation is essential. It may however also suggest the need to intervene earlier prior to the development of psychosocial consequences of obesity in formative adolescent years.

The Teen-LABS consortium has also shown the effects of adolescent bariatric surgery on other organ systems. A significant number of severely obese adolescents have evidence of early kidney dysfunction [52], which appears to improve after surgery in their cohort [53]. Further work by this group demonstrated significant improvements in musculoskeletal pain and mobility [54] which they previously correlated to quality of life [55]. In addition, a range of carcinomas has also been independently associated with obesity including breast, colorectal, endometrial, hepatic and pancreatic [56]. As obesity has become a phenomenon over the last 30 years, it is possible we are yet to reach the peak of this effect on cancer incidence. Although there are no current data on this from the adolescent population, adult literature shows a 30–80% reduction in cancer risk after bariatric surgery over 10 years.

## Side Effects and Complications

Despite very encouraging results in both weight loss and resolution of co-morbidities comparable to adult series, surgical and nutritional complications need to be carefully considered when assessing the justification for adolescent bariatric surgery. A recent review of the literature by Beamish et al. compiled side effects from the four main registries of bariatric surgery in adolescents: Teen-LABS, AMOS, Saudi Arabia and the Germany Obesity Registry. Only one 30-day mortality has been reported in more than 750 cases compared to an incidence of 0.08–0.31 in adult studies. Reporting of ‘minor complications’ and additional operations appeared to be higher in adolescent studies compared to adult series with 13–17% requiring additional operations as compared to 6–7% in adult cohorts [57•].

Perioperative outcomes in 77 RYGB adolescent patients from one US centre (mean age 16.8 +/−2.1) reported 3% intraoperative complications and 22% perioperative complications (<30 days) [58]. These most commonly included gastro-jejunal anastomotic stricture (17%), reoperation (13%), anastomotic leak (7%) and dehydration (7%). The FABS-5+ and AMOS studies have contributed significantly to our understanding of long-term complications. Further procedures were common in both cohorts; 26% required endoscopy in the FABS-5+ group; however, the need for this was not reported in the AMOS cohort. Rates of intra-abdominal surgery were comparable between the FABS-5+ and AMOS groups at 24 and 25%, respectively. Whilst the FABS-5+ group had a higher need for cholecystectomy (21 vs 11%), only two patients (3%) required a diagnostic laparoscopy. The need for laparoscopy for small bowel obstruction was significantly higher in the AMOS group, required in 14%. This was comparable to a Swedish adult cohort, and they have since changed their practice to close the mesenteric window as a consequence of this finding.

The AMOS study showed that the cumulative hospital stay over 5 years was greater in the adolescent surgical patients than in their age-matched controls (16.1 vs. 2.8 days). Two patients did not survive at 9 and 24 months post-operatively in the FABS-5+ group; however, both cases were felt to be unrelated to the surgery. There was no mortality over 5 years in the AMOS group. This data indicates the risks of surgery need to be thoroughly considered and discussed with patients and their families when considering surgical weight-loss procedures.

In the absence of supplementation, inadequate absorption of calcium, vitamin D, iron, vitamin B1, B6, B12, A and folate can occur resulting in nutritional deficiencies [59, 60]. This can manifest clinically as peripheral neuropathy (inadequate B12), beriberi syndrome (B1 deficiency), iron deficiency anaemia and osteoporosis and osteopenia. Overall, 72% had a variation of nutritional deficiency in the AMOS study with ‘several’ vitamin deficiencies identified in the FABS-5+ study, which was not further quantified. In both groups, these were predominantly mild and manageable. Anaemia was found in 46% of patients in FABS 5+ with a higher transfusion rate (5 vs 2.5%) than that found in AMOS, where only 32% were affected. The AMOS 5-year study showed that although vitamin D insufficiency was 63% in surgical group 5 years post-operatively, it was 57% in adolescent controls; however, neither study had any growth velocity data. An early study published in 1994 demonstrated only 14% of adolescents undergoing RYGB took recommended supplements [61]. This emphasises the importance of the role of the nutritionists and psychologists post-operatively to encourage compliance in this group of patients.

## Cost

Though bariatric surgery clearly incurs a substantial cost at delivery, in light of the potential resolution of long-term morbidity emerging from prospective studies, it appears this cost is justified. An analysis by Teen-LABS compared the cost of adolescents with no surgery with those undergoing surgery, including rates of perioperative mortality, complications and initial morbidity over time. After 3 years, surgery led to a gain of 0.199 quality-adjusted life years (QALY) compared with no surgery at a cost of $154,684 per QALY [62]. The trajectory showed cost decreased over time, and surgery was found to be cost effective at 5 years. Although not measured, the indirect cost must be much greater considering the improvements in psychosocial health and mobility beginning to emerge from the teen-LABS group.

## Conclusions

Epidemiologic data has confirmed that paediatric obesity is increasing in prevalence and severity. Increasing studies have shown that obesity in the adolescent age group increases mortality due to a multitude of associated co-morbidities.

Multiple studies have now shown reproducible, safe and effective results of adolescent bariatric surgery with weight loss and resolution of co-morbidities equivalent to that seen in the adult population. Overall health and psychosocial well-being also appears to be improved. As long-term outcomes are still largely unknown and complications such as vitamin deficiency and re-operation appear to be slightly higher than that seen in the adult cohort, it is imperative that surgery should only be provided by multi-disciplinary specialist teams dedicated to the holistic care of paediatric patients to ensure safety and delivery of excellent clinical care.
